# Clinical and Molecular Genetic Analysis in Three Children with Wolfram Syndrome: A Novel WFS1 Mutation (c.2534T>A)

**DOI:** 10.4274/jcrpe.2894

**Published:** 2017-03-01

**Authors:** Gamze Çelmeli, Doğa Türkkahraman, Yusuf Çürek, Jayne Houghton, Sema Akçurin, İffet Bircan

**Affiliations:** 1 Akdeniz University Faculty of Medicine, Department of Pediatric Endocrinology, Antalya, Turkey; 2 Antalya Training and Research Hospital, Clinic of Pediatric Endocrinology, Antalya, Turkey; 3 University of Exeter Medical School, Institute of Biomedical and Clinical Science, Exeter, United Kingdom

**Keywords:** Wolfram syndrome, WFS1 gene, genetic testing

## Abstract

Wolfram syndrome (WS) is an autosomal recessive disorder caused by mutations in *WFS1* gene. The clinical features include diabetes insipidus, diabetes mellitus (DM), optic atrophy, deafness, and other variable clinical manifestations. In this paper, we present the clinical and genetic characteristics of 3 WS patients from 3 unrelated Turkish families. Clinical characteristics of the patients and the age of onset of symptoms were quite different in each pedigree. The first two cases developed all symptoms of the disease in their first decade of life. The heterozygous father of case 2 was symptomatic with bilateral deafness. The first ocular finding of one patient (patient 3) was bilateral cataract which was accompanying DM as a first feature of the syndrome. In this patient’s family, there were two members with features suggestive of WS. Previously known homozygous mutations, c.460+1G>A in intron 4 and c.1885C>T in exon 8, were identified in these cases. A novel homozygous c.2534T>A mutation was also detected in the exon 8 of WFS1 gene. Because of the rarity and heterogeneity of WS, detection of specific and nonspecific clinical signs including ocular findings and family history in non-autoimmune, insulinopenic diabetes cases should lead to a tentative diagnosis of WS. Genetic testing is required to confirm the diagnosis.

**WHAT IS ALREADY KNOWN ON THIS TOPIC?**

Wolfram syndrome is a rare, autosomal recessive disorder with heterogeneous clinical features including diabetes insipidus, diabetes mellitus, optic atrophy, deafness, and other manifestations. Over two hundred mutations have been reported in WFS1 gene.

**WHAT THIS STUDY ADDS?**

This article presents clinical characteristics and mutation analysis results of 3 cases with Wolfram syndrome. Since Wolfram syndrome is a rare syndrome, the present article will expand the mutation database and help to understand the disease phenotype.

## INTRODUCTION

Wolfram syndrome (WS), also named diabetes insipidus, diabetes mellitus, optic atrophy, and deafness (DIDMOAD) syndrome, is a rare autosomal recessive disorder. The main clinical features of the syndrome, namely, diabetes insipidus (DI), diabetes mellitus (DM), optic atrophy (OA) and deafness (D), constitute the elements of the acronym DIDMOAD. Other common manifestations of the syndrome are urinary tract abnormalities (neurogenic bladder, hydroureteronephrosis), hypogonadism, progressive neurodegenerative diseases (ataxia, dementia), and psychiatric problems (1). The prevalence of the disease was estimated to be 1 in 770.000 individuals and 1 in 500.000 children less than 15 years old. However, the prevalence is reported to be as high as 1 in 68.000 in the Lebanese population where consanguineous marriages are common (2).

The syndrome is caused by a loss-of-function mutation in the *WFS1* gene which is located on chromosome 4p16.1 and consists of eight exons (1). *WFS1* gene encodes wolframin, an endoglycosidase H-sensitive transmembrane glycoprotein localized in the endoplasmic reticulum (ER). Wolframin has a hydrophilic amino-terminus in cytosol and a carboxyl-terminus in the ER lumen. Wolframin is mainly expressed in pancreas, brain, heart, and muscle tissues. It plays a crucial role in the ability of ER to process and fold new proteins properly by regulating intracellular Ca^2+^ homeostasis (3).

WS is a progressive neurodegenerative disorder with a high mortality rate. The median age of death is around 30 years (range 25-49 years) (4). Clinical suspicion at an early stage is important for prompt diagnosis and proper management.

The aim of this paper was to present clinical and genetic characteristics of the syndrome as observed in 3 WS cases from 3 unrelated Turkish families, of which one had a novel homozygous missense mutation (c.2534T>A) and the other two had a previously described mutation.

## CASE REPORTS

### Case 1

A 7.8-year-old female patient first presented to our pediatric endocrinology clinic with a diagnosis of type 1 DM, bilateral sensorineural deafness, and bilateral optic atrophy. The patient had been on insulin therapy for 10 months. Her parents were first cousins. Pancreatic autoantibodies including anti-glutamic acid decarboxylase antibody, anti-insulin antibody and anti-islet cell antibody, as well as thyroid and celiac antibodies were all negative.

When she was 10.4 years old, the patient was diagnosed to have partial central DI, detected by the water deprivation and desmopressin challenge test (DCT). At this time, the anterior pituitary gland was found to be of normal size and structure on cranial magnetic resonance imaging (MRI). Genitourinary tract ultrasound (US) revealed bilateral mild pelvicalyceal dilatation and severe distension of bladder. Post-void residual urine was 73 mL (high for age), and there was no sensation of urination indicating neurogenic bladder. Sublingual desmopressin replacement therapy was initiated.

Sequence analysis of the *WFS1* gene was performed, and a previously known homozygous splicing mutation (c.460+1G>A) was found in intron 4 (5,6,7). Her parents were heterozygous for the same mutation.

### Case 2

An 8-year-old female patient was referred to our pediatric endocrinology clinic with symptoms of diabetic ketoacidosis. Her mother and father were first cousins. Her father has been suffering from bilateral deafness.

In further evaluations, pancreatic, thyroid, and celiac antibodies were found to be negative. Due to the continuation of polyuria and polydipsia in a state of normoglycemia, DCT was performed. A diagnosis of central DI was considered. Urodynamic examinations showed incomplete emptying of the bladder. Bilateral mild pelvicalyceal dilatation was detected in urinary US.

At age 10 years, the patient presented with complaints of decreased visual acuity and loss of color vision. Optic nerve atrophy was detected on ophthalmoscopy and optical coherence tomography. Left-sided sensorial D was detected by audiometry.

Genetic analysis identified a previously known homozygous missense mutation (c.1885C>T) in exon 8 (7,8,9,10,11). Both her parents were heterozygous for the same missense mutation.

### Case 3

A 12.4-year-old female patient presented to our pediatric endocrinology outpatient clinic with a diagnosis of type 1 DM and a left-sided cataract diagnosed at ages 3 years and 12 years, respectively. Her parents were first cousins. Her maternal cousin had type 1 DM, DI, ataxia, and end-stage chronic renal failure due to neurogenic bladder and hydronephrosis. Another cousin had also type 1 DM, DI, and neurogenic bladder.

Left- and right-sided cataract surgery was performed at ages 12.5 and 13 years, respectively.

At age 16 years, partial central DI was detected by DCT. Bright spot was not present on the imaging of neurohypophysis by MRI. Bilateral sensorineural D was detected by audiometry. At age 19 years, bilateral OA was detected by routine ophthalmoscopic examination. When she was 20 years old, gabapentin was prescribed because of bilateral neuropathic pain along the peroneal nerves. Antidepressant therapy was given for a state of minor depression. Grade 2 hydronephrosis was detected when the patient was 24 years old.

**Figure 1:**
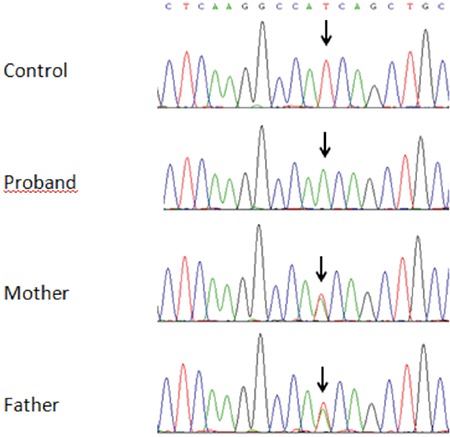
Electropherograms of mutant alleles in WFS1 gene. The proband is homozygous for T to A transversion at nucleotide position 2534 (c.2534T>A) in exon 8 of WFS1 causing isoleucine to be replaced by asparagine at codon 845 (p.I845N). Both parents are heterozygous for the same mutation (patient 3)

Genetic analysis of the *WFS1* gene revealed a novel homozygous missense mutation in exon 8. Homozygous T to A exchange at nucleotide position 2534 (c.2534T>A) leads to an isoleucine to asparagine exchange at codon 845 (p.I845N) (Figure 1). Her mother and father were heterozygous for the same mutation. Her maternal cousins did not agree to a genetic analysis.

Clinical features and genetic analysis of the patients are shown in Table 1.

## DISCUSSION

WS is a rare neurodegenerative disorder. Juvenile-onset DM and OA are the prominent and earliest features of the disease in the pediatric age group (1). Only 14-58% of the patients have all four components of DIDMOAD (4,12).

Insulin-dependent, non-autoimmune DM is often the first manifestation of WS which presents at an average age of 6 years (range from 3 weeks to 16 years) (1,4). It was shown that *WFS1* mutation increases ER stress, triggers the apoptotic pathway causing progressive β-cell loss, and impairs insulin secretion by disrupting intracellular Ca2+ homeostasis (13). When compared with type 1 DM, WS patients had a lower daily insulin requirement, lower hemoglobin A1c values, and a decreased tendency to develop ketoacidosis, findings attributed to the maintenance of some residual pancreatic β-cell mass (2,14). In the present cases, the age range at onset of diabetes was consistent with the literature. Only one of the patients (case 2) was admitted with ketoacidosis, which is a less common condition.

OA, a consistent finding in all patients, occurs at an average age of 11 years (range from 6 weeks to 19 years) with reduced visual acuity and loss of color vision (2). It is important to screen all patients with type 1 DM for OA to enable an early diagnosis of WS (1). Other less frequent ocular abnormalities reported are cataract (29.6-66.6%), pigmentary retinopathy (30%), diabetic retinopathy (7.6-34.6%), pigmentary maculopathy, glaucoma, abnormal pupillary light reflexes, and nystagmus (1,14). It is striking that cataract can be the first ocular finding and that OA can develop later, as was the case in one of our patients (case 3). Although DM and OA association is the best diagnostic criterion for WS, WS should also be suspected in patients with non-autoimmune, insulin-deficient DM and in patients with atypical ocular abnormalities such as cataract.

In WS patients, central type DI becomes apparent in the second decade of life, at an average age of 14 years (range from 3 months to 40 years), with a frequency of 51-87%. Previous studies have shown that gliosis, atrophy, and functional defects can be present in hypothalamic paraventricular and supraoptic nuclei (2).

Slowly progressive high-frequency sensorineural D is seen in 62% of WS patients at an average age of 16 years (range 5-39 years) (4). Animal studies have shown wolframin expression involving ion homeostasis in inner ear cells (15). Audiometric testing enables an early diagnosis of sensorineural deafness.

Urinary tract abnormalities (neurogenic bladder, hydroureteronephrosis, and recurrent infections) are common findings (58%) in WS, with a median age of onset of 20 years (range 10-44 years). Two of our patients developed neurogenic bladder and pelvicalyceal dilation in their first decade of life. Also, a maternal cousin of patient 3 was found to have end-stage chronic renal failure, secondary to neurogenic bladder and hydronephrosis, as a severe finding. Another cousin was reported to have neurogenic bladder. As renal failure is one of the important causes of death in WS, a careful assessment for urinary tract abnormalities and urinary infections are recommended (1,4).

In WS, neurological complications are reported to appear at a median age of 30 years (5-44 years) in 62% of the cases. The most common symptom is truncal ataxia. Other common neurological signs are loss of gag reflex, loss of olfaction, myoclonus, epilepsy, nystagmus, and central apnea. The median age of death is 30 years (25-49 years) mostly due to neurological complications especially central respiratory apnea secondary to brain stem atrophy (2,4). MRI scans demonstrate generalized brain atrophy of visual pathways, cerebellum, brainstem, and cerebral cortex (1). One of our 3 patients (case 3) developed bilateral neuropathic pain in the legs at age 20 years. One of her maternal cousins who did not consent to molecular analysis had ataxia.

Psychiatric disease and behavioral disorders (severe depression, psychosis, organic brain syndrome, and impulsive verbal and physical aggression) are reported in 60% of WS patients (16). Because of the high incidence of suicidal behaviors, psychiatric consultation and follow-up is an essential part of the treatment of these patients (1). Minor depression was diagnosed in one of our three patients (case 3).

Other clinical findings, such as hypogonadism, pituitary hormone deficiency, gastrointestinal manifestations, and cardiac defects were not detected in our patients.

To date, over two hundred mutations, with a wide spectrum, have been reported in WS patients from different ethnic groups. In many patients, loss-of-function mutations such as stop, frame-shift, and splice site mutations were found. Missense mutations were reported in 35 % of the cases (8). Although there is no clear genotype/phenotype correlation, it was shown that harboring mutations other than missense ones lead to more severe disease and earlier onset of DM and OA (3).

In our second case, a previously reported homozygous missense mutation (c.1885C>T) in *WFS1* gene was found (7,8,9,10,11). In this pedigree, which was heterozygous for the mutation (p.R629W), bilateral D was present in the father. Kadayifci et al (9), who had first described this mutation, have reported that sensorineural D can be present in heterozygous carriers for *WFS1* gene mutation. It has been shown that heterozygous carriers in a WS family have an increased risk of the manifestations of WS, especially sensorineural deafness, psychiatric illness, and DM (9,10,17).

A novel homozygous missense mutation was identified in our third patient. The isoleucine residue at codon 845 is highly conserved across species and it is therefore likely that the p.I845N mutation is pathogenic. In silico analysis using sorting intolerant from tolerant and polymorphism phenotyping v2 predicted that the mutation affects the protein function and causes the damage.

In conclusion, by presenting these 3 patients, we would like to emphasize that WS is a clinically heterogeneous disease. Its clinical signs can be seen at any age and its many features may escape from attention. As early diagnosis is important in order to handle the treatable complications of WS, routine ophthalmoscopic and urological evaluation is recommended in all patients with non-autoimmune, insulin-deficient DM at the time of diagnosis. A diagnosis of WS should be suspected in diabetic patients with uncommon associations such as D and ocular findings. Genetic mutation analysis plays a key role for definitive diagnosis and allows carrier detection.

## Figures and Tables

**Table 1:**
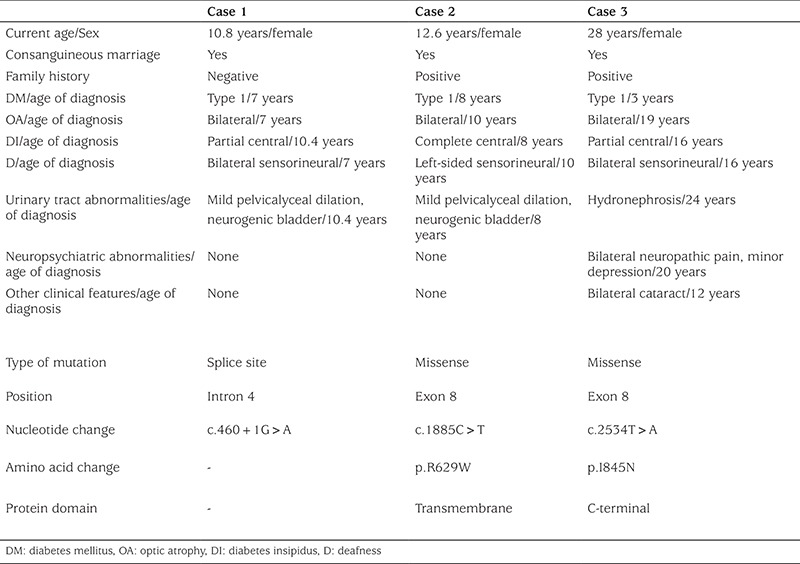
Clinical and genetic features of the patients with Wolfram syndrome
